# Infección por *Aspergillus flavus* y *Rhizopus oryzae complex* en paciente con diabetes mellitus

**DOI:** 10.7705/biomedica.6358

**Published:** 2023-03-30

**Authors:** María Alejandra Pérez, Luisa Martínez, Juan Bravo, Brenda Rodríguez, Paola Quintero, Pablo Moncada

**Affiliations:** 1 Departamento de Infectología, Fundación Valle del Lili, Cali, Colombia Departamento de Infectología Fundación Valle del Lili Cali Colombia; 2 Departamento de Microbiología, Fundación Valle del Lili, Cali, Colombia Departamento de Microbiología Fundación Valle del Lili Cali Colombia; 3 Departamento de Patología, Fundación Valle del Lili, Cali, Colombia Departamento de Patología Fundación Valle del Lili Cali Colombia; 4 Departamento de Medicina Interna, Fundación Valle del Lili, Cali, Colombia Departamento de Medicina Interna Fundación Valle del Lili Cali Colombia

**Keywords:** Aspergillus flavus, Rhizopus oryzae, mucormicosis, aspergilosis, infecciones fúngicas invasoras, sinusitis, diabetes mellitus, Aspergillus flavus, Rhizopus oryzae, mucormycosis, aspergillosis, invasive fungal infections, sinusitis, diabetes mellitus

## Abstract

La sinusitis micótica es una condición patológica que puede presentarse en pacientes con diabetes mellitus y estar asociada a una crisis hiperglucémica. Es una entidad agresiva con complicaciones locales que incluyen afectación de la órbita y el sistema nervioso central, y compromiso vascular. A pesar del tratamiento quirúrgico y antimicótico, la mortalidad es de hasta el 75 %.

Se describe el caso de una paciente con diagnóstico de cetoacidosis diabética y signos de oftalmoplejía unilateral que llevaron al estudio con resonancia magnética del sistema nervioso central; se encontraron signos de sinusitis, meningitis y cerebritis. Los estudios microbiológicos iniciales fueron negativos, y los biomarcadores galactomanano sérico y el antígeno de *Cryptococcus* también fueron negativos. Tras el manejo quirúrgico, se llegó a la identificación de *Aspergillus flavus* y *Rhizopus* spp. en el tejido de los senos paranasales. La paciente recibió tratamiento con posaconazol y, tras dos meses de seguimiento, había presentado mejoría clínica. La infección fúngica dual y la infección por *A. flavus* son entidades poco frecuentes y de relevancia clínica, sin casos presentados previamente en nuestro país por lo que este corresponde a un caso de interés clínico.

## Abstract (en)

Fungal sinusitis is a pathology that can occur in patients with diabetes mellitus and be associated with a hyperglycemic crisis. It is an aggressive entity with local complications that include involvement of the orbit or the central nervous system, and vascular involvement. Despite surgical and antifungal treatment, mortality raises up to 75%. We report the case of a female patient with a diagnosis of diabetic ketoacidosis and signs of unilateral ophthalmoplegia, which led to the study with magnetic resonance imaging of the central nervous system, finding signs of sinusitis, meningitis, and cerebritis. Initial microbiological studies were negative, and biomarkers such as serum galactomannan and *Cryptococcus* antigen were also negative. After surgical management and the identification of *Aspergillus flavus* and *Rhizopus* spp. in sinus tissue, the patient received treatment with posaconazole and after two months of follow-up she presented clinical improvement. Dual fungal infection and infection by A. flavus are uncommon and clinically relevant entities, with no cases previously reported in our country, therefore this corresponds to a case of clinical interest.

Las infecciones fúngicas invasivas corresponden a una condición patológica que amenaza la vida con una mortalidad del 21 al 80 % y se han descrito más frecuentemente en pacientes inmunosuprimidos ^1^. Aunque no se refiere de forma rutinaria a los pacientes con diagnóstico de diabetes mellitus como inmunosuprimidos, este grupo de población presenta alteraciones en los mecanismos de inmunidad, como la reducción de los linfocitos T, la disfunción en la actividad de los neutrófilos con aumento de apoptosis de los mismos y la reducción de citocinas inflamatorias, que los pone en riesgo de presentar una infección fúngica invasiva ^1^^,^^2^.

En el estudio de Borjian, *et al*. ^3^, se diagnosticó diabetes mellitus en el 36 % de 490 pacientes con infecciones fúngicas invasivas, y las entidades más frecuente fueron las neoplasias hematológicas y el trasplante de órgano sólido; sin embargo, en un metaanálisis que incluyó 851 pacientes, se encontró como la condición subyacente más frecuente en el 40 % de los casos ^4^. De igual manera, en los pacientes con diabetes mellitus, se ha descrito una mayor prevalencia de colonización fúngica (34 % en comparación con 4,7 % en pacientes no diabéticos) ^5^.

La infección fúngica invasiva más frecuente es la causada por levaduras del género Candida spp. (46,7 %), seguida de las infecciones por hongos filamentosos (42 %); la localización más usual de las infecciones fúngicas es el pulmón, seguido de los senos paranasales ^6^. En este último caso, puede haber o no invasión de la mucosa, lo cual determina el riesgo de extensión ósea y al sistema nervioso central ^7^^,^^8^.

Los agentes etiológicos más implicados en la infección fúngica de los senos paranasales son los hongos de la clase Zigomicetes en el 58 % de los casos, seguidos de *Aspergillus* spp. en el 29 % ^1^; puede presentarse coinfección en 4 a 9,4 % de las infecciones ^1^^,^^9^. Conviene resaltar que el término mucormicosis se refiere a las infecciones causadas por hongos del orden de los mucorales, los agentes patógenos más frecuentes son *Rhizopus* spp., *Mucor* spp. y *Lichteimia* spp. ^10^.

En este tipo de infecciones, la mortalidad se ha asociado a la extensión de la infección fúngica, siendo del 34 % en las infecciones localizadas y del 75 % en la infección rinocerebral ^4^. El diagnóstico temprano es fundamental para evitar esta complicación y otras consecuencias también temidas, como los eventos isquémicos y la diseminación al sistema nervioso central ^8^.

En la clasificación de rinosinusitis fúngica invasiva se han descrito como características de importancia la duración de la enfermedad menor de cuatro semanas y los signos de invasión en la histopatología, teniendo en cuenta que la extensión de la necrosis e inflamación puede variar dependiendo del tipo de inmunosupresión. Otras formas de presentación de las infecciones fúngicas rinosinusales incluyen la invasión con granulomatosis, la bola fúngica y la rinitis eosinofílica ^11^.

Los síntomas descritos en la sinusitis fúngica incluyen dolor facial, fiebre y rinorrea. En la infección avanzada, pocos pacientes presentan síntomas asociados con infección sinusoidal y son más frecuentes los síntomas asociados a invasión de otras estructuras anatómicas como diplopía, cefalea, alteración visual y neurológica ^8^.

Al realizar el estudio imagenológico de estos pacientes, la resonancia magnética es la mejor opción para la evaluación de los senos paranasales y la diseminación a tejidos adyacentes; los patrones, como ausencia de realce, se asocian a un peor pronóstico al relacionarse con alta carga fúngica y necrosis ^12^.

Las infecciones diseminadas a otros órganos pueden ocurrir en 15 a 23 % de los casos ^13^, por lo que se recomienda la práctica de endoscopia para descartar el compromiso gastrointestinal. De igual manera, con el objeto de evaluar otras áreas de , se indica la realización de imágenes de tórax, abdomen y cráneo ^10^. En los pacientes inmunocompetentes o con diagnóstico de diabetes mellitus en la infección por mucorales, las lesiones cutáneas y el compromiso rinoorbital son las presentaciones asociadas más usuales, por lo que las lesiones en piel, senos paranasales y órbitas deben buscarse de forma sistemática ^10^.

La confirmación requiere de biopsia, la cual tiene una sensibilidad del 75 al 85 % si se toma del cornete medio que corresponde a la zona de mayor afectación; otras áreas que se pueden incluir en la toma de la muestra incluyen el tabique nasal y el piso de la cavidad nasal ^14^.

En el estudio histopatológico de la infección por mucorales, se observan hifas no tabicadas o paucitabicadas con invasión tisular en las tinciones de hematoxilina y eosina, ácido peryódico de Schiff y Gomori Grocott. A partir del rendimiento descrito del estudio patológico, es conveniente realizar cultivos para la identificación de género y especie ^10^. Entre los criterios diagnósticos para las infecciones fúngicas invasivas probadas, se encuentra el examen histopatológico, la demostración de infección fúngica en un tejido estéril o la recuperación de estructuras fúngicas en muestras obtenidas en un procedimiento estéril o mediante hemocultivos ^6^.

El manejo de los pacientes con sinusitis fúngica invasiva tiene varios componentes: estabilización de la condición de base, desbridamiento y terapia antifúngica ^8^. En la revisión de Roden, *et al*. ^15^, de 929 casos con mucormicosis, las tasas de supervivencia fueron del 57 % cuando se hizo manejo quirúrgico, del 61 % cuando se administró anfotericina B como monoterapia y del 70 % en el manejo dual quirúrgico y antifúngico. De manera análoga, en los pacientes diabéticos con mucormicosis, la supervivencia también ha sido mayor en el grupo de pacientes con terapia dual que en aquellos que solo reciben tratamiento antifúngico (74 % *Vs*. 51 %) ^16^.

A pesar de la relevancia del manejo quirúrgico, la cirugía se practica solo en el 65 % de los pacientes inmunocompetentes y el 21,4 % de los pacientes hematológicos ^17^. Al Según el conocimiento de los autores, no existe un estudio que compare las técnicas quirúrgicas usadas para el manejo de mucormicosis, pero, en efecto, el desbridamiento radical hasta obtener bordes limpios se recomienda para preservar la órbita y mejorar los resultados clínicos ^18^^,^^19^.

La terapia antifúngica de primera línea es la anfotericina B liposómica, si bien se ha reportado que dosis altas de 10 mg/kg/día pueden aumentar la tasa de mejoría, también se ha asociado con un aumento sustancial en el valor de creatinina sérica, por lo que en ausencia de compromiso del sistema nervioso central se consideran de elección las dosis intermedias de 5 mg/kg/ día. Otros fármacos como el isavuconazol y el posaconazol han demostrado ser igualmente efectivos, pero son considerados líneas de salvamento y no deberían utilizarse como primera aproximación terapéutica ^20^. Algunos antifúngicos nuevos podrían tener utilidad en el tratamiento de esta infección y aún se encuentran en investigación, como el fosmanogepix, dirigido contra la proteína Gtw1 (proteína esencial para el tráfico y anclaje de las proteínas a la pared celular del hongo), y nuevos azoles, como el PC1244, e inhibidores de CYP, como el VT-1161 .

El tratamiento se recomienda hasta que la condición de inmunosupresión se haya resuelto y se tenga resolución en las imágenes diagnósticas, lo cual requiere un seguimiento clínico estrecho e individualización de los casos para determinar la extensión del esquema terapéutico ^10^.

## Presentación de caso

Se describe el caso de una paciente de sexo femenino y de 48 años con diagnóstico de diabetes mellitus de tipo 2 en tratamiento con insulina, quien ingresa al servicio de urgencias por cuatro días de evolución con alteración del estado de conciencia.

Durante su evaluación se documentaron criterios de cetoacidosis diabética grave y, en los gases arteriales, se encontró un pH menor de 8 y valor no calculable de HCO_3_ (bicarbonato), lo que requirió manejo en la unidad de cuidados intensivos; en los análisis séricos, el valor de la hemoglobina glucosilada fue del 12,8 % y, en el hemograma, se encontró leucocitosis (28.680 células/μl) con neutrofilia (24.320 células/μl) como hallazgo por resaltar; otros estudios se muestran en el cuadro 1.

**Cuadro 1:** Valores de laboratorio al ingreso de la paciente a la unidad de cuidados intensivos

Parámetro		Ingreso a la unidad de cuidados intensivos
Leucocitos (células/pl)		28.680
	Neutrófilos	24.320
	Linfocitos	1.030
	Monocitos	1.900
Hemoglobina (g/dl)		14,7
Volumen corpuscular medio (fl)		87,9
Hemoglobina corpouscular media (pg)		30,1
Ancho de distribución eritrocitaria (%)		12,8
Hematocrito (%)		42,9
Plaquetas (células/μl)		407.000
Creatinina (mg/dl)		1
BUN (mg/dl)		20,6
Sodio (mmol/L)		137
Glucemia (mg/dl)		462
Potasio (mmol/L)		3,9
Cloro (mmol/L)		107
Calcio (mmol/L)		1,26
PCR (mg/dl)		3,72
Gases arteriales		pH > 6,8 pCO_2_: 20 mm Hg HCO3: no calculable BE: no calculable
		pO_2_: 333 mm Hg PaO_2_/FiO_2_: 512
Lactato (mmol/L)		0,7

En el examen físico en la unidad de cuidados intensivos, se encontró anisocoria izquierda no reactiva por lo que se ordenó una tomografía simple de cráneo la cual no demostró lesiones isquémicas ni hemorrágicas , ante la sospecha de un evento isquémico en el sistema nervioso central, se ordenó una resonancia magnética (RM). En este estudio, se identificaron signos de meningitis y ocupación del seno esfenoidal izquierdo, ante lo cual se obtuvo una muestra de líquido cefalorraquídeo que no demostró alteraciones en el estudio citoquímico ni microbiológico. De esta manera, se inició tratamiento antibiótico con vancomicina y ceftriaxona, bajo el diagnóstico de meningitis y sinusitis.

**Figura 1:**
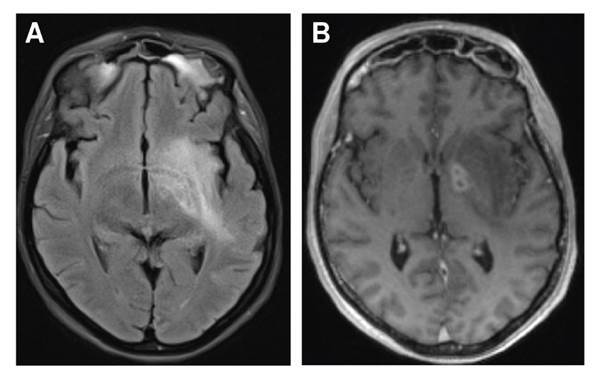
Focos de cerebritis en el ganglio basal en secuencia FLAIR (A) y T1 después del contraste (B)

A pesar del manejo antibiótico, 10 días después del ingreso al hospital la paciente presentó hemiparesia izquierda, desviación de la comisura labial con parálisis facial central izquierda y disartria. Se practicó una nueva RM en la que se evidenciaron hallazgos de sinusitis fúngica invasiva con compromiso del espacio masticador, el *cavum* de Meckel, la fosa craneal media y la órbita izquierda; además, hubo signos de cerebritis y meningitis con trombosis del seno cavernoso izquierdo (figura 1).

**Figura 2:**
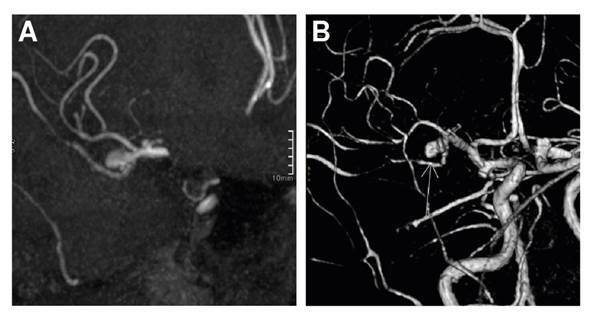
Aneurisma sacular en secuencias en tiempo de vuelo MIP (A) y 3D (B)

Asimismo, la paciente fue valorada por oftalmología y, en la valoración del fondo de ojo, se encontraron signos de neuropatía óptica isquémica anterior no arterítica y oclusión de la rama de la arteria retinal temporal superior en el ojo izquierdo. Ante los hallazgos de compromiso vascular, se practicó una angiorresonancia magnética en la que se observaron aneurismas saculares de múltiples territorios vasculares: en la arteria cerebral media derecha, la carótida interna y el segmento comunicante de la arteria carótida interna derecha (figura 2).

Teniendo en cuenta los hallazgos previamente reportados, se consideró que la paciente cursaba con infección fúngica invasiva; se realizaron de forma paralela pruebas de galactomanano sérico, antígeno urinario de *Histoplasma* y antígeno sérico de *Cryptococcus*, los cuales fueron negativos. Dada la alta probabilidad clínica de infección fúngica invasiva, se inició tratamiento con anfotericina B liposómica a dosis de 5 mg/kg/ día; además, a la paciente se le practicó antrostomía. Respecto al manejo de las formaciones aneurismáticas, teniendo en cuenta el tamaño y la localización, no se indicó su manejo endovascular. En el procedimiento quirúrgico realizado por otorrinolaringología se evidenció tejido necrótico en los cornetes, los senos etmoidales y en los esfenoidales.

**Figura 3:**
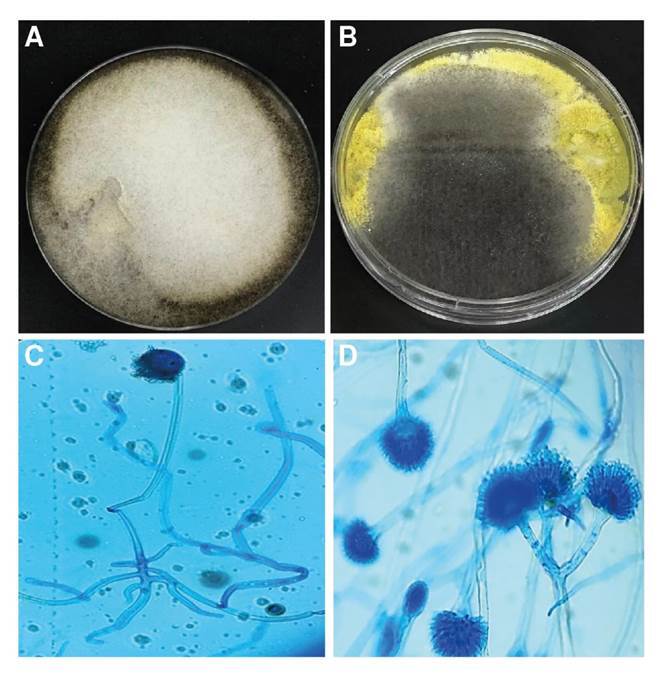
Crecimiento mixto de hongos filamentosos en agar Sabouraud. Colonia verde corresponde *Aspergillus flavus/orizae* y colonia gris *Rizophus orizae* (A) (B) Tinción de azul de lactofenol. *Rhizopus oryzae* (C) *Aspergillus flavus* (D) con objetivo de 40X.

**Figura 4:**
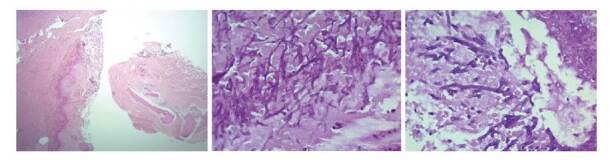
(A) Tejido conectivo denso y óseo desvitalizado con dos formas de hongos filamentosos (H&E, 4X) , uno con hifas delgadas septadas ramificadas (B) y otro con hifas hialinas anchas sin septos con objetivo de 40X (C)

En el examen directo con KOH al 10 % de las muestras de tejido de los senos paranasales, fue posible observar hifas no tabicadas sugestivas de zigomycetos, y en los cultivos en agar Sabouraud incubados a 26 °C se obtuvo crecimiento mixto de dos hongos filamentosos hialinos (figura 3) que fueron identificados por técnica proteómica con el equipo MALDI- TOF MS como *Aspergillus flavus/oryzae* y *Rhizopus oryzae* complex. Estas estructuras fúngicas también se describieron en las biopsias con tinción de PAS y Gomori-Grocott (figura 4).

**Figura 5:**
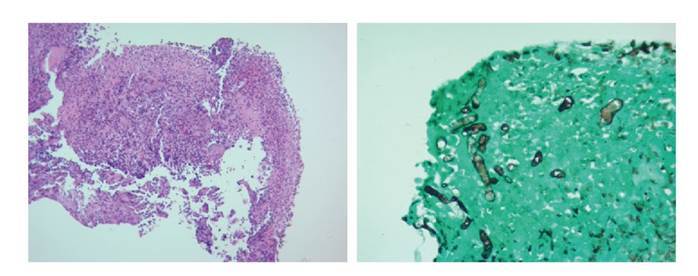
(A) Mucosa gástrica ulcerada con una inflamación crónica granulomatosa (H&E, 10X), asociada a un hongo filamentoso con hifas hialinas anchas no septadas (coloración de Gomory 40X) (B).

La paciente cursó con hemorragia de las vías digestivas altas durante la hospitalización, por lo que se le hizo una endoscopia de vías digestivas en la cual se evidenciaron abundantes coágulos con mucosa gástrica ulcerada y nodular; la biopsia se reportó como gastritis crónica e inflamación aguda grave, ulcerada, con identificación de estructuras fúngicas en las tinciones (figura 5).

En la evaluación del compromiso infeccioso diseminado, se obtuvo una tomografía de tórax en la cual se apreciaron lesiones nodulares difusas y, en el lavado broncoalveolar, los estudios microbiológicos fueron negativos. En la tomografía de abdomen no se encontraron signos de procesos infecciosos y en el ecocardiograma no hubo hallazgos que sugirieran endocarditis infecciosa.

La paciente completó 14 días de tratamiento con anfotericina B liposómica y, posteriormente, se inició el tratamiento con posaconazol a dosis diarias de 300 mg, que se ha extendido de forma indefinida; para el momento del reporte, completaba dos meses de tratamiento con posaconazol sin signos clínicos que sugirieran una nueva infección invasiva y no se había realizado aún el control imagenológico.

### Consideraciones éticas

Se mantuvo la confidencialidad de la información del paciente, así como su autorización.

## Discusión

El caso presentado es poco habitual al tratarse de una infección fúngica por *Aspergillus* spp. y por *Rhizopus* spp.; la identificación de la especie A. flavus también corresponde a un hallazgo inusual. Las presentaciones clínicas de la infección por este último incluyen infecciones broncopulmonares, orbitarias, cutáneas, de senos paranasales y óseas ^21^. En los pacientes inmunocompetentes, *A. flavus* se ha descrito como la causa más común de rinosinusitis fúngica granulomatosa, y *A. fumigatus* es la etiología usual en las formas invasivas del compromiso sinusoidal ^22^.

En la búsqueda de otros casos de sinusitis invasiva en pacientes inmunocompetentes por A. flavus, se encontró un caso reportado también en Cali ^23^. En dos estudios adicionales se reportan casos de *A. flavus* sin descripción de las características clínicas ni del estado inmunológico de los pacientes, cuatro casos reportados por Singh, *et al*. ^24^, y tres casos más por Krishnan, *et al*. ^25^. En el estudio de Lao, *et al*. ^6^, de pacientes con diabetes mellitus, la prevalencia de *A. flavus* fue del 5,4 % como agente causal de la infección fúngica invasiva, incluyendo tanto infecciones por mohos como por levaduras en cualquier órgano, mientras que *A. fumigatus* tuvo una prevalencia del 12,6 %. Por el contrario, en el estudio de Jeong, *et al*., *Rhizopus oryzae* fue el agente etiológico más frecuente de la mucormicosis, responsable del 33 % de los casos reportados ^4^.

En Colombia, se han descrito 60 casos de infección por zigomicetos, 12 de los cuales tenían como agente etiológico a *Rhizopus* spp. ^26^. Sin embargo, la coinfección fúngica por *A. flavus y Rhizopus* spp. es una entidad poco frecuente sin casos reportados previamente en nuestro país, por lo que este corresponde a un caso de interés clínico.

Es destacable la presencia de aneurismas en la angiorresonancia cerebral de la paciente. Aunque no fue posible comprobarlo, en el estudio histopatológico podrían corresponder a verdaderos aneurismas micóticos. Los aneurismas micóticos representan el 5 % de los aneurismas cerebrales y se han asociado al diagnóstico de diabetes mellitus ^27^. La etiología fúngica en los mismos es infrecuente; se han reportado casos de infección por *Aspergillus* spp., *Candida* spp. y zigomicetos, siendo *Aspergillus* spp. el hongo más común ya que, al producir invasión vascular, las hifas pueden generar trombosis y daño endotelial ^28^. El manejo quirúrgico se indica en pacientes con efecto de masa y ruptura; para los demás casos, la elección de tratamiento endovascular depende de la localización y el tamaño de las lesiones ^29^.

En la infección por *Aspergillus* spp., otras complicaciones del sistema nervioso central incluyen meningitis, abscesos cerebrales, empiema subdural y sangrado intraparenquimatoso ^28^. El diagnóstico se basa en la biopsia de cerebro o en el estudio de tejido adyacente, y los cultivos de líquido cefalorraquídeo y PCR son métodos diagnósticos también utilizados. La frecuencia de alteración en el líquido cefalorraquídeo no ha sido reportada. Sin embargo, en los casos con compromiso del sistema nervioso central, se describe como inusual ^30^, lo cual explicaría la ausencia de alteración en el citoquímico de la paciente pese a los hallazgos imagenológicos de meningitis y cerebritis.

En la búsqueda de condiciones que correspondieran a inmunosupresión, la paciente no presentaba signos ni síntomas que sugirieran autoinmunidad ni neoplasia hematológica; la prueba ELISA para HIV fue negativa, y la medición de inmunoglobulinas y el valor de linfocitos fueron normales. Ante el diagnóstico de infección fúngica invasiva, es importante tener una fuerte sospecha clínica de inmunodeficiencia primaria en ausencia de tratamiento inmunosupresor.

En el caso de infección por hongos filamentosos, las inmunodeficiencias más asociadas son el síndrome de neutropenia congénita, las mutaciones en el gen *STAT1* y la haploinsuficiencia de factor 2 unido a GATA ^31^. Las mutaciones con localizaciones específicas incluyen la deficiencia de STAT3 (asociada a aspergilosis en sistema nervioso central y sinoorbitaria) y la deficiencia de CARD9 (asociada a aspergilosis invasiva del sistema nervioso central y abdominal) ^31^. Considerando la gran frecuencia de infección fúngica invasiva en la población con diabetes mellitus, no se realizaron pruebas genéticas durante la hospitalización de la paciente.

## Conclusión

La infección fúngica invasiva debe considerase en los pacientes con diabetes mellitus, en particular, en aquellos que presentan crisis hiperglucémicas. El tratamiento antifúngico temprano y el desbridamiento quirúrgico extenso son los pilares del tratamiento de estos pacientes. Además, el control metabólico adecuado y la corrección de condiciones como la anemia y la hipoglucemia pudieran mejorar el pronóstico ^6^.
